# HPLC-ESI-QTOF-MS/MS-Guided Profiling of Bioactive Compounds in Fresh and Stored Saffron Corms Reveals Potent Anticancer Activity Against Colorectal Cancer

**DOI:** 10.3390/ph19010149

**Published:** 2026-01-14

**Authors:** Sanae Baddaoui, Ennouamane Saalaoui, Oussama Khibech, Diego Salagre, Álvaro Fernández-Ochoa, Samira Mamri, Nahida Aktary, Muntajin Rahman, Amama Rani, Abdeslam Asehraou, Bonglee Kim, Ahmad Agil

**Affiliations:** 1 Laboratory of Bioresources, Biotechnology, Ethnopharmacology and Health, Faculty of Sciences, Mohammed First University, Bd Mohamed VI BP 717, Oujda 60000, Morocco; 2 Laboratory of Applied Chemistry and Environment (LCAE), Faculty of Science, Mohammed First University, Bd Mohamed VI BP 717, Oujda 60000, Morocco; 3 Department of Pharmacology, BioHealth Institute Granada (IBs Granada), Neuroscience Institute (CIBM), School of Medicine, University of Granada, 18016 Granada, Spainaagil@ugr.es (A.A.); 4 Department of Analytical Chemistry, Faculty of Sciences, University of Granada, 18011 Granada, Spain; 5 Department of Pathology, College of Korean Medicine, Kyung Hee University, Seoul 02447, Republic of Korea; 6 Korean Medicine-Based Drug Repositioning Cancer Research Center, College of Korean Medicine, Kyung Hee University, Seoul 02447, Republic of Korea

**Keywords:** *Crocus sativus*, corms, anthraquinones, anticancer, HPLC-ESI-QTOF-MS/MS, colorectal cancer

## Abstract

**Background**: Saffron (*Crocus sativus* L.) corms, often discarded as agricultural by-products, are a promising and sustainable source of bioactive metabolites with potential therapeutic relevance. However, their anticancer potential remains largely underinvestigated. **Objectives**: This study aimed to compare the phytochemical composition of hydroethanolic extracts from fresh (HEEF) and stored (HEES) saffron corms and to evaluate their anticancer effectiveness against colorectal cancer cells. **Methods**: Phytochemical profiling was performed using HPLC-ESI-QTOF-MS/MS. Cytotoxicity against T84 and SW480 colorectal cancer cell lines was determined by the crystal violet assay. Apoptosis-related protein modulation was assessed by Western blotting. Additionally, molecular docking, molecular dynamics simulations, and MM/GBSA calculations were used to investigate ligand–target binding affinities and stability. **Results**: Both extracts contained diverse primary and secondary metabolites, including phenolic acids, flavonoids, triterpenoids, lignans, anthraquinones, carotenoids, sugars, and fatty acids. HEES showed higher relative abundance of key bioactive metabolites than HEEF, which was enriched mainly in primary metabolites. HEES showed significantly greater dose-dependent cytotoxicity, particularly against SW480 cells after 24 h (IC_50_ = 34.85 ± 3.35). Apoptosis induction was confirmed through increased expression of caspase-9 and p53 in T84 cells. In silico studies revealed strong and stable interactions of major metabolites, especially 3,8-dihydroxy-1-methylanthraquinone-2-carboxylic acid with COX2 and crocetin with VEGFR2. **Conclusions**: Stored saffron corms possess a richer bioactive profile and show enhanced anticancer effects in vitro compared with fresh saffron corms, suggesting that they may represent a promising source of compounds for the future development of colorectal cancer therapeutics.

## 1. Introduction

Colorectal cancer (CRC) is the third most prevalent malignancy worldwide and ranks as the second leading cause of cancer-related deaths [1,2]. In 2023, more than 153,000 new colorectal cancer (CRC) cases were diagnosed in the United States, making it the third most common cancer. Men are more likely than women to develop CRC (53%) [3]. Despite significant advancements in early diagnosis and treatment, CRC remains a leading cause of cancer-related mortality [4]. The disease arises from multiple risk factors, including age, family history, inherited colon cancer-related mutations, dietary habits, and environmental variables. Diets rich in red and processed meat, excessive alcohol consumption, obesity, overconsumption of foods heavy in fat or sugar, insulin resistance, and cigarette smoking are associated with an increased risk. In contrast, physical activity and diets rich in calcium, fiber, garlic, and milk have been shown to exert protective effects against colorectal cancer [3,5].

Current CRC treatment strategies include surgery, immunotherapy, radiotherapy, chemotherapy, and targeted therapy. Among these, chemotherapy remains the most common approach for advanced stages [6]. However, it is often limited by severe side effects and the emergence of chemoresistance [7]. Consequently, there is increasing interest in phytotherapy, a treatment strategy based on bioactive molecules derived from medicinal plants [8,9,10]. Numerous studies have demonstrated the anticancer potential of plant-based compounds against various malignancies, including CRC [11].

*Crocus sativus* L. (Saffron), a medicinal and aromatic plant, has been traditionally used for centuries to treat various diseases, including cancer. For example, Lahmass et al (2017), [12] reported significant anticancer activity of saffron by-products against A549 lung carcinoma and U373 glioblastoma cells. Despite this, most research and cultivation efforts focus on saffron stigma, the highly valued spice, while its by-products, such as corms, remain underexplored. Interestingly, corms have been reported to exhibit anticancer activity against HeLa cancer cells and cervical cancer cells [13,14,15]. In addition, they possess immunomodulatory, antifungal, and antibacterial effects [16,17], metal-chelating and antioxidant properties [17,18,19,20,21], and anti-inflammatory properties, including the ability to suppress pro-inflammatory cytokines [22]. Advancements in technology have greatly impacted pharmacological research by combining computational and experimental approaches to improve health care. Molecular docking, in particular, has enhanced the identification of bioactive compounds and the exploration of potential therapeutic targets [23].

Corms are typically kept as planting materials in post-harvest storage for extended periods, and this phase is often associated with shifts in primary versus secondary metabolism. Comparing fresh- and stored-corm batches can therefore provide an initial view of how post-harvest handling may relate to chemical composition and downstream bioactivity; however, comparisons across different harvest years may also capture inter-annual environmental variability and should be interpreted with appropriate caution.

The present study aims to perform a comparative analysis of stored and fresh corms of *Crocus sativus*, focusing on phytochemical composition and anticancer activity against colorectal cancer cells. Although several studies have reported anticancer effects of saffron preparations against colorectal cancer cells, most have focused on stigmas and on a limited set of known constituents, such as crocin, crocetin, and safranal [24,25,26,27]. In contrast, our work targets saffron corms, an underutilized by-product, and explicitly compares fresh and one-year stored corms by integrating high-resolution HPLC ESI QTOF MS/MS profiling, in silico ADME triage, multitarget molecular docking, molecular dynamics simulations, MM/GBSA calculations, and in vitro cytotoxicity and protein-expression assays. We specifically investigated the SW480 and T84 colorectal cancer cell lines: SW480 cells originate from a primary colorectal adenocarcinoma, whereas T84 cells originate from a metastatic colorectal carcinoma; using these two lines, therefore, allows us to assess whether the extracts are active across distinct molecular and phenotypic subtypes of colorectal cancer. We provide a comprehensive view of how storage-induced changes in the corm metabolome relate to multi-pathway anticancer effects in colorectal cancer cells.

## 2. Results

### 2.1. Phytochemistry Analysis

**Figure 1:**
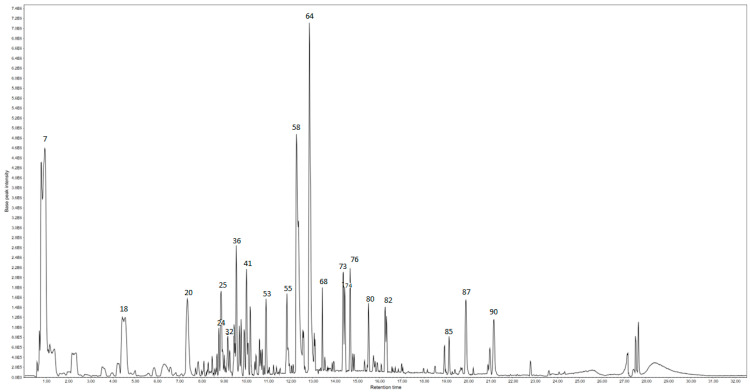
Base peak chromatogram of the hydroethanolic extract (HEES) of stored *Crocus sativus* corms.

**Figure 2:**
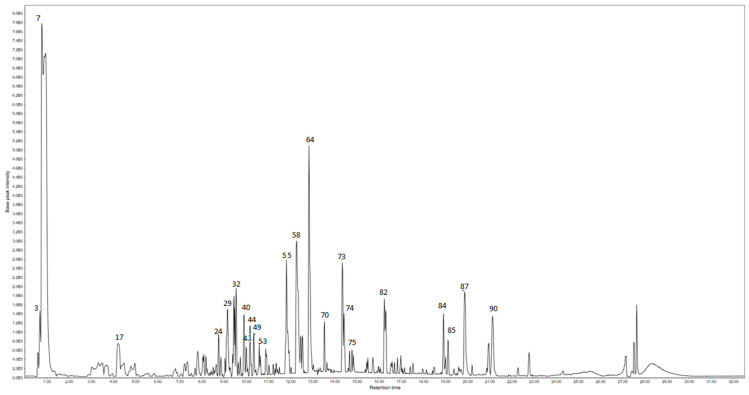
Base peak chromatogram of the hydroethanolic extract (HEEF) of fresh *Crocus sativus* corms.

HPLC-ESI-QTOF-MS/MS analysis (Table A1 (Appendix A), Figure 1 and Figure 2) revealed that both fresh (HEEF) and stored (HEES) corms of *Crocus sativus* contain a wide spectrum of primary and secondary metabolites, including carbohydrates, organic acids, lipids, phenolic compounds, flavonoids, anthraquinones, lignans, and glycosides. Despite this shared chemical diversity, clear compositional shifts were observed between the two types of extracts, reflecting metabolic adjustments occurring during storage.

Carbohydrates and organic acids: Fresh corms (HEEF) were characterized by a higher abundance of simple carbohydrates such as sucrose and fructose, together with elevated levels of citric acid and other tricarboxylic intermediates. This pattern reflects an active primary metabolism and energy turnover in freshly harvested tissues. In contrast, stored corms (HEES) exhibited lower levels of free sugars and a relative increase in sugar oxidation and degradation products (e.g., arabinoic and phthalic acids), suggesting reduced respiratory activity and a shift toward catabolic processes during storage. The accumulation of dicarboxylic and hydroxylated organic acids in HEES also indicates the onset of oxidative metabolism and partial degradation of cellular constituents.

Lipids and fatty acid derivatives: The lipid profile of both extracts revealed the presence of saturated and unsaturated fatty acids. Fresh corms showed relatively balanced levels of palmitic, oleic, linolenic, and rumenic acids, consistent with intact membrane structures and stored energy reserves. In contrast, stored corms showed higher levels of oxidized and hydroxylated fatty acids, such as hydroxyoctadecadienoic and dihydroxyoctadecanoic acids. This shift toward oxidized lipid derivatives suggests that progressive lipid peroxidation and oxidative modification occurred during post-harvest storage, although causality cannot be assigned in the present design.

Phenolic compounds (including flavonoids, coumarins, and lignans): Phenolic metabolites represented one of the most diverse chemical groups detected in both extracts. Fresh corms contained higher levels of reactive phenolic compounds, such as simple phenols, hydroxycinnamic acids, and glycosylated flavonoids, which are typically associated with growth and primary defense mechanisms. Conversely, stored corms accumulated more oxidized and structurally stable phenolic derivatives, including hydroxybenzoic and phthalic acid derivatives, coumarins, lignans, and other conjugated aromatic compounds. This pattern is consistent with oxidative transformation and differential preservation of phenolics during storage, leading to enrichment of more stable aromatic structures.

Anthraquinones and related quinones: A pronounced increase in anthraquinone-type compounds was observed in the stored corms (HEES). These metabolites, such as various anthracenecarboxylic acid derivatives and hydroxyanthraquinones, were more abundant or exclusively detected in HEES, consistent with storage-associated oxidative transformation and stabilization of phenolic precursors during post-harvest handling. Anthraquinones are known to arise through oxidative transformations of precursor phenolics and polyketide pathways, suggesting that prolonged storage triggers secondary metabolic reorganization and stress-related biosynthesis. Fresh corms, on the other hand, contained fewer quinone-type compounds, consistent with their more reduced and metabolically active state.

Glycosides, lignans, and terpenoid derivatives: Both extracts contained glycosidic and terpenoid derivatives, but their relative abundance varied. Fresh corms exhibited a broader range of glycosides linked to sugars and simple phenolics, whereas stored corms showed a greater presence of complex terpenoid and lignan derivatives. This compositional shift supports the idea that storage favors the stabilization and accumulation of secondary metabolites with lower metabolic turnover and greater structural rigidity.

The comparative phytochemical profile indicates that fresh corms (HEEF) are dominated by primary metabolites and reactive phenolic compounds associated with active metabolism and growth, whereas stored corms (HEES) show a clear enrichment in oxidized, aromatic, and quinone-type secondary metabolites. These changes reflect a metabolic transition from energy production and biosynthesis toward oxidative stabilization and metabolite transformation processes during storage.

### 2.2. SwissADME Profiling of the Nine Major Compounds Common to Both Extracts

Early ADME (Absorption, Distribution, Metabolism, and Excretion) screening is essential to ensure that in silico hits have a realistic chance of translating into cellular activity and, ultimately, drug-like behavior. We therefore prioritized the nine major constituents that are either shared by both extracts or markedly enriched in at least one of them (Table 1): 7-hydroxycoumarin-6-carboxylic acid, Azelaic acid, Rhamnalpinogenin, Endocrocin, 3,8-dihydroxy-1-methylanthraquinone-2-carboxylic acid, Cinnalutein, Crocetin, Aloesaponarin I, and Citric acid. This panel captures both common and storage-enhanced chemotypes likely to contribute to the biological readouts. Other detected constituents, including compounds such as gingerol and chrysophanol, with reported anticancer activity, were not included in this first in silico panel in order to limit computational complexity; they remain attractive candidates for future docking and MD studies. By profiling their physicochemical properties and potential DMPK (drug metabolism and pharmacokinetics) liabilities using the SwissADME platform, we aimed to pre-filter structurally diverse candidates with promising pharmacological relevance. This approach increases the reliability of downstream molecular docking by focusing on ligands with tractable permeability, solubility, and metabolic risk, thereby improving the credibility of the binding hypothesis [28].

Collectively, the set sits well inside an oral drug-like envelope: molecular weight spans 188–342 g/mol, with cLogP from −1.51 to 2.28, TPSA of 63.6–132 Å^2^, 1–4 HBDs, 4–7 HBAs, and 1–9 rotatable bonds; all compounds have 0 Lipinski and 0 Veber violations, consistent with acceptable passive permeability and conformational restraint. Bioavailability scores cluster at 0.56 (typical for polar acids/polyphenols), with Azelaic acid standing out at 0.85 (low MW 188.22 g/mol, TPSA 74.6 Å^2^, CLogP 1.49), indicating the most favorable oral exposure in the panel; Aloesaponarin I is slightly lower at 0.55, despite having the highest lipophilicity (CLogP 2.28), suggesting a solubility–permeability trade-off [29]. Crocetin combines the lowest TPSA (63.6 Å^2^) with the highest flexibility (nine rotatable bonds) and moderate CLogP (1.87), a profile compatible with good membrane transit and even potential BBB permeability (TPSA < 90 Å^2^), though flexibility may modestly reduce docking pose precision. At the polar extreme, Citric acid (TPSA 132 Å^2^, CLogP −1.51) and Endocrocin (TPSA 132.13 Å^2^) are near the upper TPSA limit for efficient passive absorption and are unlikely to cross the BBB without transport assistance or formulation; nevertheless, both remain within the Lipinski/Veber space. Predicted CYP liabilities are reassuring for CYP2D6 (none flagged), whereas ~45% are predicted as CYP3A4 inhibitors, specifically Rhamnalpinogenin, Endocrocin, Cinnalutein, Crocetin, and Aloesaponarin I, which warrants attention for drug–drug interaction risk and metabolic clearance during hit selection. Finally, the prevalence of carboxylate/phenolic motifs implies partial or full deprotonation at physiological pH, favoring strong ionic/H-bonding to Lys/Arg and polar residues in protein pockets; this is advantageous for target engagement but can temper passive permeability, in line with the predominantly 0.56 bioavailability scores. Overall, Azelaic acid and Crocetin emerge as the most balanced for oral exposure, while Citric acid and polyhydroxylated anthraquinones (e.g., Endocrocin) may require formulation or prodrug strategies if they advance beyond docking.

**Figure 3:**
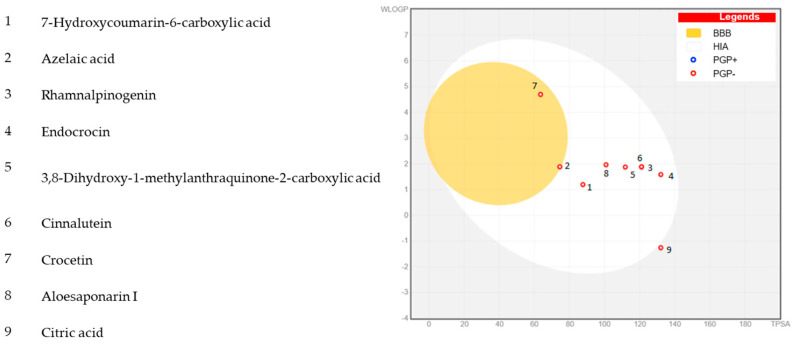
BOILED-Egg plot (WLOGP vs. TPSA) for the 9 major compounds: predicted HIA, BBB penetration, and P-gp status.

In Figure 3 (BOILED-Egg), most of the nine compounds fall within the white ellipse (HIA), indicating a high likelihood of passive intestinal absorption [30]. Compounds 7 and 2 sit in the yellow yolk (BBB), suggesting potential brain penetration, whereas compound 9 lies outside the HIA ellipse (TPSA ≈ 132 Å^2^, WLOGP ≈ −1.5), consistent with limited passive uptake; compound 4 is near this high-polarity boundary as well. Compounds 3 and 6 overlap at the same position because they share identical TPSA and WLOGP values, implying the same ADME prognosis. All markers are PGP- (red-rimmed), so no P-gp efflux liability is predicted in this model.

Importantly, these SwissADME outputs reflect predicted in vivo oral exposure (intestinal absorption and first-pass liabilities) and therefore are not expected to correlate directly with the cell-based cytotoxicity results obtained under direct cellular exposure conditions.

### 2.3. Cytotoxic Effect on SW480 and T84 Colon Cancer Cells

**Figure 4:**
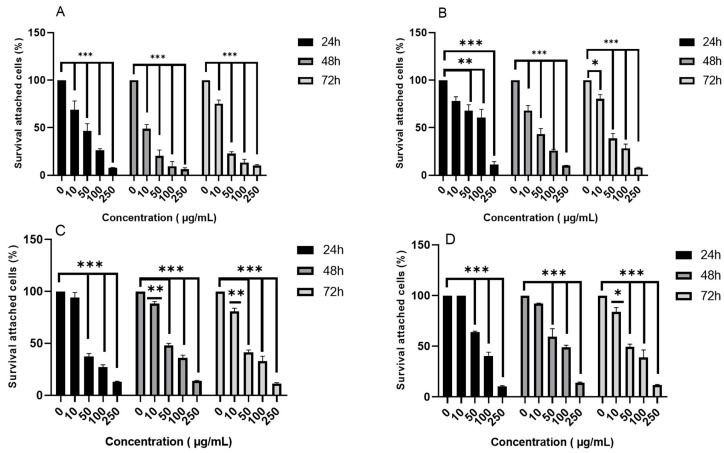
Cytotoxic effects of HEES (**A**,**C**) and HEEF (**B**,**D**) on the colon cancer cell lines SW480 (**A**,**B**) and T84 (**C**,**D**), respectively, determined using the crystal violet assay at different concentrations of the extract (10, 50, 100, 250 µg/mL). HEEF: hydroethanolic extract from fresh corms. HEES: hydroethanolic extract from stored corms. Data are expressed as mean ± SEM. Statistical significance compared with the control group (0 µg/mL) is indicated as * *p* < 0.05, ** *p* < 0.01, and *** *p* < 0.001.

This study highlights the anticancer effect of *Crocus sativus* corms against the colon cancer cell lines T84 and SW480. The results for the % of surviving attached cells evaluated by the crystal violet assay indicated that both HEES and HEEF displayed clear dose-dependent reductions in cell viability over the 10–250 μg/mL range in SW480 and T84 cells (Figure 4). At 10 μg/mL, time-dependent effects between 24, 48, and 72 h were modest, particularly for HEES on SW480 cells, whereas at 50–250 μg/mL, a progressive decrease in viability over time became more evident. Thus, concentration appears to be the main driver of the response, with additional time-dependent effects at higher doses.

The IC50 doses are illustrated in Table 2. At 24 h, the IC50 for SW480 cells was significantly lower for HEES (34.85 ± 3.35 µg/mL) compared to HEEF (186.60 ± 1.14 µg/mL), indicating markedly higher activity (*p* < 0.001). For HEES in SW480 cells, the IC_50_ decreased further at 48 h (17.18 ± 0.88 µg/mL), followed by a slight increase at 72 h (23.83 ± 0.37 µg/mL); however, statistical analysis showed that this increase between 48 h and 72 h was not significant. For T84 cells, the IC50 values for HEES and HEEF were closer, suggesting comparable efficacy in this cell line.

### 2.4. Western Blotting Results

**Figure 5:**
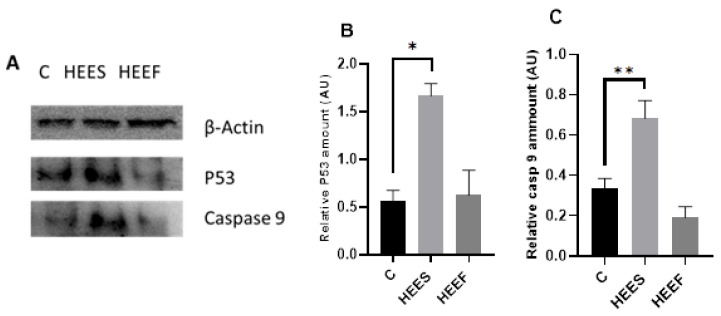
Effects of *Crocus sativus* corm extracts on p53 and caspase-9 protein expression in T84 colon cancer cells. (**A**) Representative Western blot images showing p53 and caspase-9 protein expression in T84 cells after treatment with HEES and HEEF. (**B**) Quantitative analysis of p53 protein expression levels normalized to the loading control. (**C**) Quantitative analysis of caspase-9 protein expression levels normalized to the loading control. C: control; HEES: hydroethanolic extract of stored corms; HEEF: hydroethanolic extract of fresh corms; caspase-9: casp-9. Data are expressed as mean ± SEM. Statistical significance compared with the control group (0 µg/mL) is indicated as * *p* < 0.05, ** *p* < 0.01.

The results of protein expression in T84 after treatment with HEES and HEEF are illustrated in Figure 5. HEES treatment was associated with an increased expression of the apoptotic markers p53 (Figure 5B) and caspase-9 (Figure 5C) in T84 cells compared to HEEF, whereas HEEF did not significantly alter these proteins. These data suggest that HEES may engage apoptotic pathways, while HEEF’s effects on cell viability may involve growth inhibition or alternative, non-apoptotic mechanisms.

In the HEEF-treated group, the expression levels of p53 and caspase-9 were comparable to those of the control, suggesting that the HEEF-induced reduction in cell viability is not primarily mediated by activation of the p53–caspase-9 intrinsic apoptotic pathway under the tested conditions.

This observation indicates that alternative mechanisms may be involved, such as p53-independent growth arrest, non-apoptotic cell death pathways, or modulation of other cancer-related signaling cascades. For this reason, we explored additional possible mechanisms using molecular docking and molecular dynamics simulations, targeting proteins involved in cell cycle regulation, inflammation, angiogenesis, and survival signaling. This integrative in vitro–in silico approach was intended to generate mechanistic hypotheses that could account for the biological effects of HEEF beyond classical intrinsic apoptosis.

### 2.5. Molecular Docking

Molecular docking is a critical bridge between our SwissADME triage and wet-lab validation: by testing shape/electrostatic complementarity and estimating binding free energy, it lets us prioritize which of the nine major constituents are most likely to engage disease-relevant pockets and modulate pathways we already probed experimentally (p53/caspase-9). In this panel, the chosen structures exemplify complementary cancer axes: caspase-9 (1NW9) captures the initiator caspase in an XIAP-BIR3-regulated state, directly linked to intrinsic apoptosis; CDK2 (2FVD) reflects cell cycle control; COX-2 (5KIR) addresses the inflammation–tumorigenesis interface; EGFR kinase (1M14) and VEGFR2 kinase (3VO3) cover growth-factor signaling and angiogenesis, respectively; MMP-7 (7WXX) informs on extracellular-matrix remodeling and invasion; p53 bound to DNA (1TUP) allows exploration of potential stabilization/allosteric mechanisms; and the TCF4-β-catenin complex (1JPW) targets the Wnt transcriptional hub, central to colorectal cancer [31,32,33,34]. Screening our nine compounds across these pockets will rank chemotypes by pose quality, flag target-specific opportunities (e.g., ionic/phenolic contacts versus hydrophobic clefts), and guide the identification of ligands to advance to more detailed MD or assay work for each target.

Table 3 summarizes the docking free energies (kcal/mol) of the nine major constituents across eight cancer-relevant proteins and reveals a clear enrichment of high-affinity chemotypes among anthraquinone/flavonoid scaffolds. The standout is crocetin, which shows a target-selective minimum of −9.8 kcal/mol at VEGFR2 (3VO3). A multitarget cluster comprising 3,8-dihydroxy-1-methylanthraquinone-2-carboxylic acid (down to −8.9 at CDK2/2FVD, −8.7 at COX-2/5KIR, −8.3 at EGFR/1M14, −8.0 at p53/1TUP), cinnalutein (−8.7 at 2FVD, −8.3 at 1M14), aloesaponarin I (−8.7 at 2FVD, −8.6 at 5KIR, −8.5 at 3VO3), and endocrocin (−8.6 at 2FVD, −8.2 at 5KIR/1M14), plus rhamnalpinogenin and 7-Hydroxycoumarin-6-carboxylic acid (≤−8.2 at multiple sites), consistently ranks near the top, consistent with π-π/H-bond anchoring in polar clefts [35]. In contrast, smaller aliphatic/polyacidic molecules (azelaic and citric acids) exhibit the weakest binding (mostly −4.6 to −6.1). At the target level, CDK2 (2FVD), COX-2 (5KIR), and EGFR (1M14) yield the most favorable ΔG spectra overall, whereas MMP-7 (7WXX) and TCF4-β-catenin (1JPW) return more modest affinities. Collectively, Table 1 supports prioritizing the anthraquinone/flavonoid chemotypes for multitarget optimization; in particular, 3,8-dihydroxy-1-methylanthraquinone-2-carboxylic acid emerges as a top performer at CDK2 (2FVD, −8.9 kcal/mol) and COX-2 (5KIR, ΔG ≈ −8.7 kcal/mol), while crocetin is a candidate for a VEGFR2 (3VO3)-focused path with MD/MM-GBSA refinement prior to experimental validation.

**Figure 6:**
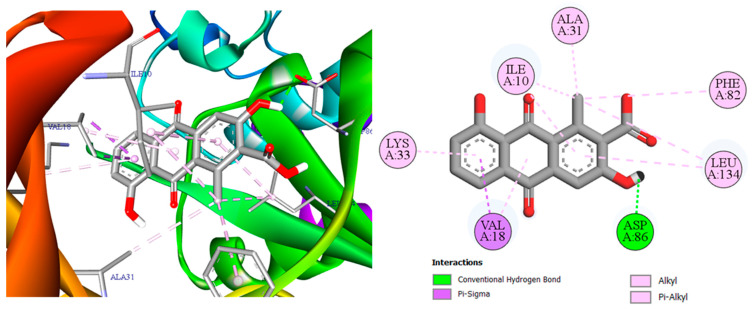
Two- and three-dimensional interaction maps of 3,8-dihydroxy-1-methylanthraquinone-2-carboxylic acid bound to CDK2 (PDB: 2FVD).

**Figure 7:**
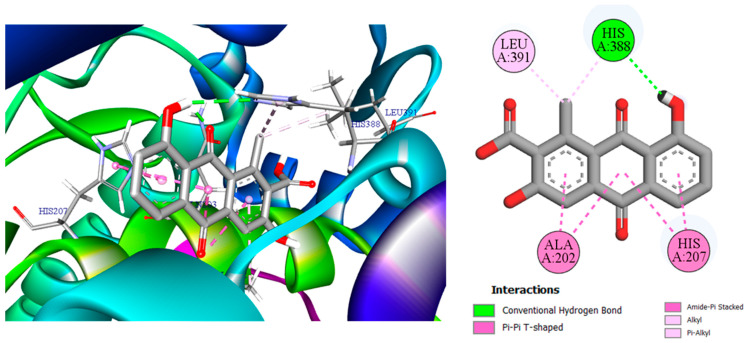
Two- and three-dimensional interaction maps of 3,8-dihydroxy-1-methylanthraquinone-2-carboxylic acid bound to COX-2 (PDB: 5KIR).

**Figure 8:**
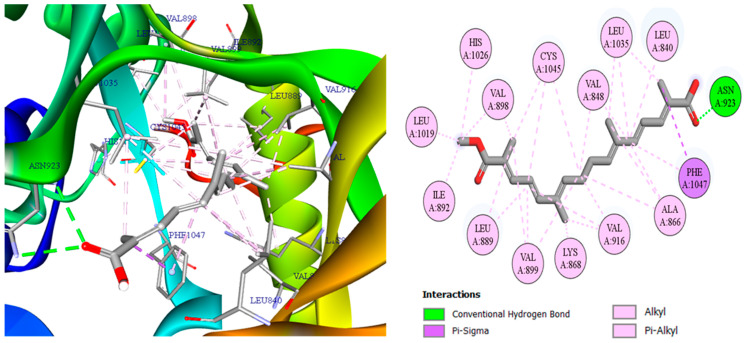
Two- and three-dimensional interaction maps of crocetin bound to VEGFR2 (PDB: 3VO3).

To explain the high affinities observed in Table 3, we turn to the 2D/3D interaction maps: Figure 6 (3,8-dihydroxy-1-methylanthraquinone-2-carboxylic acid with CDK2/2FVD), Figure 7 (the same ligand with COX-2/5KIR), and Figure 8 (crocetin with VEGFR2/3VO3). These visualizations summarize the networks of hydrogen bonds and hydrophobic contacts and will support the detailed interpretation that follows.

Figure 6 depicts a classic “anchored hydrophobe” pose of 3,8-dihydroxy-1-methylanthraquinone-2-carboxylic acid in CDK2 (2FVD). A single conventional H-bond from the terminal phenolic group to Asp86 fixes the ligand orientation, while the planar anthraquinone core is cradled by a belt of hydrophobic alkyl/π-alkyl/π-sigma contacts with Ile10, Val18, Ala31, Leu134, and Lys33, with Phe82 contributing additional aromatic–aliphatic packing. This combination of one directional polar anchor plus extensive van der Waals enclosure explains the strong docking energy reported for CDK2 [36,37]. In Figure 7, 3,8-dihydroxy-1-methylanthraquinone-2-carboxylic acid forms a single conventional H-bond with His388, providing a directional anchor. The planar anthraquinone core then engages in four aromatic contacts: two π-π T-shaped/amide-π stacked with Ala202 (backbone amide-π) and two with His207 (imidazole-π) [38,39]. In addition, two hydrophobic contacts of the alkyl/π-alkyl type are observed with Leu391 and His388. This compact network, comprising one polar anchor plus four stacking/T-shaped interactions and two hydrophobic contacts, accounts for the strong COX-2 docking energy reported in Table 3 and is consistent with the aryl-accommodating channel of the COX-2 active site.

In Figure 8, crocetin adopts a hydrophobically wrapped pose: 27 alkyl/π-alkyl contacts distribute around the entire polyene skeleton, providing extensive van der Waals enclosure that strongly stabilizes the ligand. In addition, a conventional hydrogen bond with Asn923 furnishes directional anchoring, while a π-sigma interaction with Phe1047 further consolidates the packing.

Together with Figure 6, Figure 7 and Figure 8, these results indicate two complementary binding solutions: polar-anchored hydrophobes for the anthraquinone scaffold (CDK2/COX-2) and a densely hydrophobic, lightly polarized pose for crocetin in VEGFR2. These chemotypes are prioritized for follow-up MD stability checks and targeted assays.

### 2.6. Molecular Dynamics Simulation

Molecular dynamics (MD) complements docking by testing whether a docked pose remains physically plausible once protein, solvent, and thermal motions are allowed to relax; the ligand RMSD after least-squares fitting to the protein reveals pose retention (stable complexes typically stay ≤0.3–0.4 nm), while the protein-backbone RMSF identifies which structural segments remain rigid or become flexible in response to binding [40].

**Figure 9:**
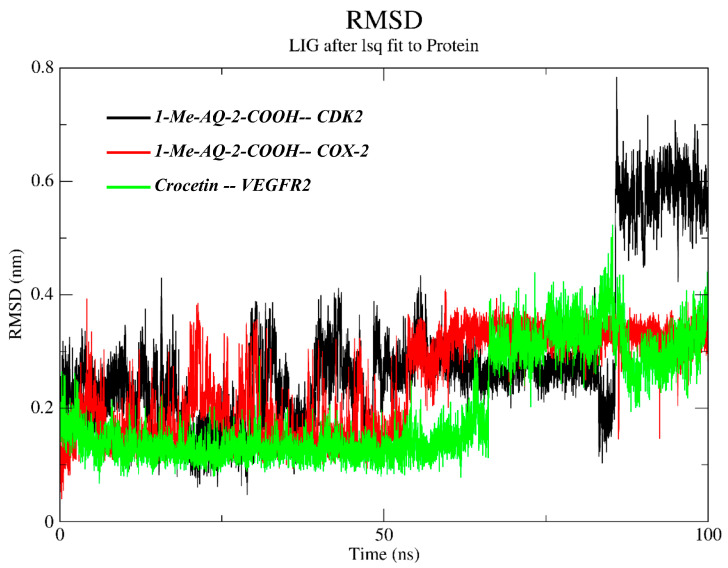
Ligand RMSD (protein-fitted) over 0–100 ns for CDK2/1-Me-AQ-2-COOH (black), COX-2/1-Me-AQ-2-COOH (red), and VEGFR2/Crocetin (green).

In our case, the RMSD traces (Figure 9) show that 1-Me-AQ-2-COOH in COX-2 (red) is the most kinetically stable: it fluctuates narrowly around ~0.3–0.35 nm across 55–100 ns. Crocetin in VEGFR2 (green) is tight up to ~60–65 ns (~0.1–0.18 nm) and then exhibits a transient excursion to ~0.40–0.5 nm before relaxing near ~0.2–0.35 nm, consistent with pocket readjustment rather than loss of binding. In contrast, 1-Me-AQ-2-COOH in CDK2 (black) is stable for ~0–85 ns (~0.15–0.30 nm) but then drifts persistently to ~0.55–0.70 nm, indicating a major reorientation/partial disengagement and, therefore, weaker kinetic stability than in COX-2 [41,42].

**Figure 10:**
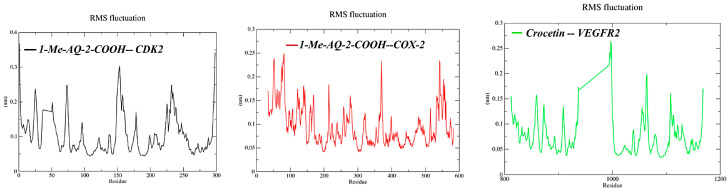
Backbone RMSF profiles (0–100 ns) for CDK2, COX-2, and VEGFR2 in complex with their ligands.

The backbone RMSF profiles (Figure 10) are largely low (<0.15–0.20 nm), with peaks confined to flexible termini/loops; COX-2 shows generally damped fluctuations with only modest loop peaks (<~0.25 nm), matching its stable ligand RMSD; VEGFR2 displays a pronounced local peak around the hinge/activation-loop region (~1000), while the kinase core remains comparatively rigid; and CDK2 exhibits elevated mobility near a loop region (~150–170) that is compatible with the late-time ligand rearrangement. Overall, MD consolidates the docking signal for COX-2 and, after relaxation, for VEGFR2, while flagging CDK2 as the least robust complex; these diagnostics justify proceeding to per-residue analysis and MM/GBSA to quantify the energetic consequences.

### 2.7. MM/GBSA Free-Energy Analysis

MM/GBSA calculations were performed for the three systems 1-Me-AQ-2-COOH/CDK2, 1-Me-AQ-2-COOH/COX-2, and Crocetin/VEGFR2 to estimate binding free energies (ΔG_bind, kcal·mol^−1^) from 100 equilibrated snapshots extracted every 0.4 ns from the 60–100 ns segment of each MD trajectory. Within the single-trajectory protocol, the binding contribution is decomposed into gas-phase molecular-mechanics terms, specifically van der Waals and Coulombic electrostatics (ΔG_gas_ = ΔE_vdW_ + ΔE_ele_), and solvent responses, comprising the polar Generalized-Born and nonpolar surface-area components (ΔG_solv_ = ΔG_GB_ + ΔG_surf_); entropy was not included unless otherwise stated. Because configurational entropy (−TΔS) was not explicitly computed (e.g., normal-mode or quasi-harmonic analysis), the MM/GBSA ΔG_bind_ values should be interpreted as relative, enthalpy-dominated estimates rather than absolute binding free energies. This analysis complements docking and MD stability metrics (RMSD/RMSF) by assigning energetic weights to the dominant interactions. As summarized in Table 4, all three systems exhibit negative ΔG_bind_ values in water, indicating thermodynamically favorable binding for 1-Me-AQ-2-COOH with CDK2 and COX-2, as well as Crocetin with VEGFR2 [43].

$$\Delta G_{\mathrm{bind}\;}=\Delta G_{\mathrm{gas}\;}+\Delta G_{\mathrm{solv}\;}=\left(\Delta E_{\mathrm{vdW}\;}+\Delta E_{\mathrm{elec}\;}\right)+\left(\Delta G_{GB}+\Delta G_{\mathrm{surf}\;}\right)\left(kcal\cdot {mol}^{-1}\right)$$

Table 4 shows that all three complexes are thermodynamically favorable in water, with a clear ranking of binding strength: Crocetin-VEGFR2 (ΔG_bind_ ≈ −44.5 kcal·mol^−1^) > 1-Me-AQ-2-COOH-COX-2 (≈−29.4) > 1-Me-AQ-2-COOH-CDK2 (≈−21.5). Across systems, the gas-phase term is strongly stabilizing and the solvation term is destabilizing, so the net affinity reflects their balance. For Crocetin-VEGFR2, the large vdW contribution (ΔE_vdW_ ≈ −50.6) and modest electrostatics (ΔE_ele_ ≈ −7.4) combine to a favorable ΔG_gas_ ≈ −58.0, while the solvation penalty is comparatively small (ΔG_GB_ ≈ +21.2) and partly offset by a sizable nonpolar term (ΔG_surf_ ≈ −7.7), yielding the most negative ΔG_bind_ [44]. In 1-Me-AQ-2-COOH-COX-2, stabilization is also vdW-dominated (ΔE_vdW_ ≈ −39.9; ΔE_ele_ ≈ −21.8; ΔG_gas_ ≈ −61.7), but the polar desolvation cost is higher (ΔG_GB_ ≈ +36.6) and the nonpolar compensation smaller (ΔG_surf_ ≈ −4.4), giving an intermediate ΔG_bind_. For 1-Me-AQ-2-COOH-CDK2, although ΔG_gas_ is the most favorable of the three (≈ −64.2) owing to stronger Coulombic stabilization (ΔE_ele_ ≈ −34.2), this advantage is largely neutralized by the largest polar solvation penalty (ΔG_GB_ ≈ +47.1) with limited hydrophobic offset (ΔG_surf_ ≈ −4.4), resulting in the weakest net affinity. Taken together, Table 4 indicates that VEGFR2 binding is driven by extensive hydrophobic burial with moderate desolvation costs, COX-2 relies on vdW contact with higher polar penalties, and CDK2 suffers the greatest desolvation penalty, despite strong gas-phase interactions consistent with the RMSD/RMSF trends observed earlier for these complexes.

### 2.8. Principal Component Analysis

Principal Component Analysis (PCA) condenses the high-dimensional MD trajectory into a few collective coordinates (PC1, PC2) that capture the largest-amplitude, slow motions (“essential dynamics”). Mapping the trajectory onto a free-energy landscape (FEL) in the PC1-PC2 plane reveals metastable basins (low-ΔG regions) corresponding to dominant conformational substates; the depth of a basin reflects thermodynamic stability, its spread reflects flexibility, and the connectivity between basins reflects possible transition pathways. PCA/FEL therefore complements RMSD/RMSF (kinetics/geometry) and MM/GBSA (ensemble-averaged thermodynamics) to validate docking under realistic dynamics [45].

**Figure 11:**
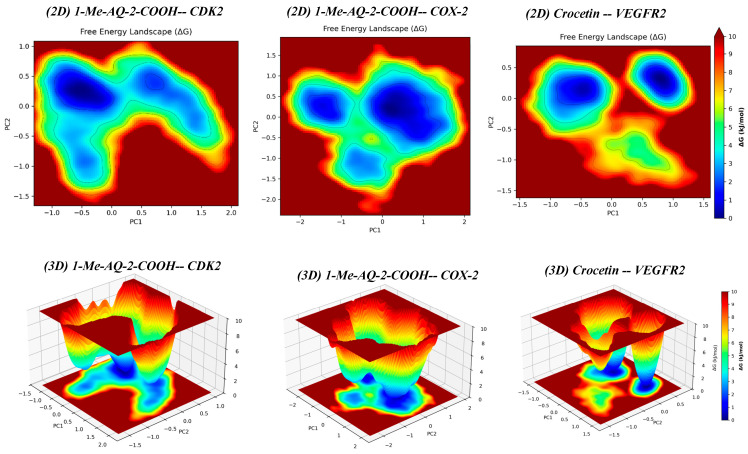
Principal component free-energy landscapes (PC1-PC2) from MD: 2D heat maps (**top**) and 3D surfaces (**bottom**) for 1-Me-AQ-2-COOH/CDK2, 1-Me-AQ-2-COOH/COX-2, and Crocetin/VEGFR2.

Figure 11 shows 2D heat maps (top) and 3D surfaces (bottom) of ΔG across PC1-PC2 for the three complexes. For 1-Me-AQ-2-COOH CDK2, the landscape is a single, broad, shallow bowl with a diffuse low-energy area, indicating one loosely defined substate and easy diffusion across nearby conformations, consistent with the late-time RMSD drift and the weakest MM/GBSA affinity (ΔG_bind ≈ −21.5 kcal·mol^−1^). Such a broad/shallow minimum implies weak confinement of the bound ensemble and therefore a less stable/less specific binding mode in which the ligand can readily reorient; this interpretation is supported by the late-time ligand RMSD drift (Figure 9), consistent with reorientation/partial disengagement, rather than a single tightly locked pose. For 1-Me-AQ-2-COOH COX-2, the FEL displays a compact dominant basin with minor shoulders, indicating a well-defined bound ensemble with limited breathing modes, matching its stable RMSD and intermediate ΔG_bind (≈−29.4 kcal·mol^−1^). For Crocetin VEGFR2, the FEL contains three basins: two deep, compact minima and one shallower basin. This does not contradict the strong GBSA affinity (ΔG_bind ≈ −44.5 kcal·mol^−1^); rather, it indicates conformational plasticity within the bound state. The two deep basins correspond to alternative, energetically equivalent packing modes of crocetin in the largely hydrophobic VEGFR2 pocket (e.g., slight tilts/rotations of the polyene chain and side-chain readjustments), while the shallow basin reflects a transient, higher-energy substate (pocket breathing/hinge motion) that also explains the brief RMSD excursion around 60–65 ns. Because occupancy concentrates in the two deep basins and the barriers between them appear to be modest (continuous blue-cyan corridors in Figure 11), the ensemble-averaged binding remains strongly favorable despite the presence of a third, less favorable region. In short, Figure 11 indicates a single loose basin for CDK2, a single tight basin for COX-2, and multiple low-energy basins for VEGFR2; this pattern aligns with the GBSA ranking and explains why crocetin achieves the most negative ΔG_bind when sampling more than one bound microstate.

## 3. Discussion

This study highlights the impact of storage on the biochemical composition and anticancer activity of *Crocus sativus* corms against colorectal cancer. HPLC-ESI-QTOF-MS/MS profiling revealed systematic differences between the batches, with the stored batch showing relative enrichment of oxidized and structurally stable secondary metabolites. These differences are consistent with post-harvest storage-associated processes, but causal attribution is limited by harvest-year confounding.

HPLC-ESI-QTOF-MS/MS analysis revealed that post-harvest storage causes major metabolic reprogramming in *Crocus sativus* corms. Fresh corms (HEEF) were dominated by primary metabolites such as simple sugars, fatty acids, and citric acid, reflecting active energy metabolism. In contrast, stored corms (HEES) showed reduced carbohydrate and TCA intermediates but higher levels of oxidized organic acids, hydroxylated fatty acids, phenolic derivatives, anthraquinones, lignans, and terpenoids. This pattern is consistent with storage-associated oxidative transformations and selective preservation/enrichment of more stable secondary metabolites during post-harvest handling. Overall, storage shifts the corm’s metabolism from growth and energy production toward oxidative stabilization and defense-related compound accumulation, helping maintain tissue viability during dormancy.

Several studies have highlighted *Crocus sativus* L. corms as a valuable source of bioactive molecules, including polyphenols such as gallic, caffeic, and salicylic acids; saponins such as oleanolic acid and azafrines 1 and 2 [15,16,21,46]; carotenoids such as crocetin [19]; and fatty acids such as linoleic acid [47]. The higher accumulation of these metabolites in stored corms may result from stress-induced synthesis triggered by oxidative or environmental factors, particularly dehydration and temperature fluctuations during storage.

Ref. [48] reported that the accumulation of secondary metabolites is regulated not only by biochemical, cellular, and developmental processes but also by abiotic stresses such as drought, salinity, temperature shifts, light, UV radiation, and nutrient limitation. In our comparison, the stored batch showed lower relative abundance of several sugars (major constituents of corms) than the fresh batch [49,50]. This pattern is consistent with carbohydrate consumption and/or transformation during dormancy and post-harvest handling, although year-to-year variability may also contribute. Similar decreases in carbohydrate levels during storage have been reported for other plant storage organs, such as onion bulbs (*Allium cepa* L.) [51], and for cereal grains during long-term storage [52].

Our findings corroborate these observations: storage-induced metabolic reprogramming favors the synthesis and accumulation of secondary metabolites such as crocetin and rhamnalpinogenin, which were absent in fresh corms. At the same time, the high abundance of sugars and fatty acids in fresh corms likely provides an energy reserve and a pool of precursors for secondary metabolite biosynthesis. Thus, environmental stresses associated with storage act as triggering signals that stimulate the production of defense-related molecules, including phenols, flavonoids, lignans, anthraquinones, and carotenoids, thereby enhancing the bioactive potential of stored corms [52,53,54].

It should be noted, however, that fresh and stored corms correspond to different harvest years (2023 and 2020, respectively). Both batches were obtained from the same cultivation area, under similar agronomic practices, and were comparable in size and appearance; nonetheless, inter-annual environmental differences may have contributed to some of the observed compositional changes. The direction and magnitude of the shifts we report for depletion of sugars and enrichment of secondary metabolites during storage are consistent with previous studies on saffron corms and other geophytes during storage [54], which supports our interpretation of our findings as primarily storage-driven. However, we cannot fully disentangle storage effects from year-to-year variability. Controlled studies using replicated harvest years and staged storage times (e.g., 0, 3, 6, 12 months) are required to establish reproducibility and causality.

The anticancer activity of hydroethanolic extracts from fresh (HEEF) and stored (HEES) *Crocus sativus* corms was assessed in the colorectal cancer cell lines SW480 and T84. The analysis addressed both direct cytotoxicity and apoptotic potential, focusing on the expression of key proteins such as p53 and caspase-9. Both extracts displayed dose-dependent cytotoxic effects; however, HEES was significantly more potent than HEEF, particularly against SW480 cells after 24 h of treatment. This enhanced activity can be attributed to storage-induced biochemical changes that promote the accumulation of secondary metabolites, notably polyphenols, flavonoids, and saponins, which are well recognized for their biological activity. Consistently, chemical profiling revealed higher concentrations of cinnalutein, endocrocin, rhamnalpinogenin, crocetin, and 3,8 dihydroxy 1 methylanthraquinone 2 carboxylic acid in HEES compared with HEEF. Their favorable docking and MM/GBSA profiles make them plausible contributors to the enhanced anticancer effects of stored corms. However, the extract is a complex mixture, and we cannot exclude significant roles of other identified or unidentified constituents, as well as synergistic or antagonistic interactions between components. Our data therefore support a correlation between storage-induced enrichment of secondary metabolites and increased in vitro anticancer activity, but they do not prove causality for individual compounds.

Comparison between cell lines revealed that SW480 cells are more sensitive to HEES than T84 cells, likely reflecting inherent genetic and biological differences. The heightened response of SW480 suggests that specific pathways in this cell type are more effectively targeted by metabolites enriched in HEES. This finding highlights the need to explore additional signaling pathways beyond p53 and caspase-9, including COX-2, CDK2, VEGFR2, EGFR, and MMP-7, which were identified as potential in silico targets. Western blot analyses confirmed that apoptosis is a central mechanism underlying the anticancer effects of the extracts. Treatment with 30 µg/mL induced significant activation of p53 and caspase-9 in T84 cells, with stronger effects observed for HEES than for HEEF. These differences correlate with chemical composition: endocrocin, abundant in HEES, exhibits high affinity for caspase-9, while rhamnalpinogenin and 3,8-dihydroxy-1-methylanthraquinone-2-carboxylic acid preferentially interact with p53, indicating that storage enriches corms with compounds capable of more effectively modulating apoptotic pathways.

It should be noted that the crystal violet assay measures the number of attached viable cells, and the observed decrease in signal is the result of both cell death and reduced proliferation. The concomitant increase in p53 and caspase 9 expression in T84 cells is compatible with activation of apoptotic pathways, but additional assays, such as Annexin V/propidium iodide staining, caspase activity measurements, and cell cycle analysis, would be required to conclusively distinguish between cytostatic and cytotoxic effects. In addition, inclusion of a reference anticancer drug (e.g., 5-fluorouracil) in future assays would allow benchmarking of extract potency across studies.

Notably, predicted ADME parameters related to absorption and first-pass effects describe systemic exposure after oral dosing, whereas the cell-based assay evaluates intrinsic antiproliferative/cytotoxic potential under direct exposure, without gastrointestinal barriers, plasma distribution constraints, or hepatic first-pass clearance. Consequently, a molecule may display strong activity in vitro despite its predicted poor oral absorption, because cellular responses depend on local concentration, intracellular accumulation, and transporter-mediated uptake, while first-pass processes are largely absent in this experimental setting. Conversely, favorable oral ADME predictions do not guarantee cellular potency. Therefore, SwissADME is used here as an early developability filter (to flag potential absorption/metabolic liabilities for future translation) rather than as a predictor of cell-based efficacy.

In silico analysis indicated that the anticancer effects of our extracts extend beyond apoptosis. Several compounds are predicted to target additional key processes in carcinogenesis, including cell proliferation (EGFR, CDK2), angiogenesis (VEGFR2), inflammation (COX-2), and tumor invasion (MMP-7). This multitarget potential contributes to the superior efficacy of the extracts, particularly HEES, and highlights anthraquinones and carotenoids as promising structural classes for therapeutic development. Among these, 3,8-dihydroxy-1-methylanthraquinone-2-carboxylic acid emerged as a strong candidate against CDK2 and COX-2, while crocetin preferentially targets VEGFR2, as confirmed by molecular dynamics simulations and MM-GBSA calculations.

3,8-Dihydroxy-1-methylanthraquinone-2-carboxylic acid represents a novel avenue for anticancer research, with additional reported activities including antimicrobial [55], antibiofilm [56], antiplasmodial [57], and neuroprotective effects, notably via inhibition of tropomyosin receptor kinase B, suggesting potential applications in neurological disorders and certain cancers [58]. Crocetin, extensively studied for its anticancer properties, inhibits colon cancer cell proliferation by inducing apoptosis and downregulating inflammation-related genes [59]. It suppresses HCT-116 cell proliferation, prevents migration, reduces VEGF and MMP-9 expression, and enhances p38 phosphorylation via the MAPK pathway [60]. In SW480 cells, crocetin (0.8 mmol/L) blocks the S phase of the cell cycle by activating p21 independently of p53 while inducing apoptosis and impairing DNA repair [61].

These observations are broadly consistent with previous studies reporting antiproliferative activity of *Crocus sativus* corm extracts in colorectal and other cancer cell lines. For example, Sánchez-Vioque et al. (2016) [62] reported that 70% ethanolic corm extracts inhibited Caco-2 cell proliferation (ED50 = 0.054 mg/mL), causing cell separation, necrosis, and reduced division. Similarly, other reports describe IC_50_ values below 100 µg/mL for certain cancer cell lines (e.g., HL-60 and HCT116) [63]. In our dataset, the stored-corm extract (HEES) produced IC_50_ values in the low tens of µg/mL in SW480 cells, whereas the fresh-corm extract (HEEF) showed higher IC_50_ values at early time points (up to 186.60 µg/mL at 24 h). Together, these findings support our conclusion that storage enhances the anticancer potential of corm extracts by qualitatively and quantitatively enriching their bioactive composition.

## 4. Materials and Methods

### 4.1. Plant Material

*Crocus sativus* corms originated from Taliouine, Morocco. One batch was collected in January 2020 and stored for 12 months in a dark, well-ventilated room at 25 ± 2 °C, with ambient relative humidity fluctuating between 40% and 50% during the storage period (Oujda, Morocco). No signs of excessive moisture or fungal contamination were observed, reproducing local agronomic post-harvest practice. A second batch was freshly collected in January 2023. In both cases, corms were handled carefully to avoid mechanical damage. At the time of extraction, the corms were in a non-sprouting state (no visible bud/leaf emergence); any sprouting corms were excluded. The species is documented in the botanical garden of Université Mohammed Premier in Oujda, Morocco, under the registration number HUMPOM210.

### 4.2. Extraction

Both fresh and stored *Crocus sativus* corms were cleaned and dried at 37 °C in a ventilated oven at 37 °C until constant weight (a mild temperature was selected to limit thermal degradation of heat-labile constituents). The dried material was then crushed with an automated grinder. For extraction, 10 g of the crushed corms was macerated with 100 mL of ethanol–water (50:50, *v*/*v*) for 24 h at 25 ± 2 °C in the dark under continuous gentle agitation on a magnetic stirring plate (350 rpm). Each extraction was performed using three successive maceration cycles on the same plant material. After each 24 h immersion, the solvent was collected and replaced with fresh solvent, and the process was repeated twice more. The three extracts were combined to maximize recovery of soluble constituents and filtered, and the solvent was removed by gentle evaporation in an oven set to 38 °C for 24 h. The resulting dry hydroethanolic extracts (HEES and HEEF) were stored at −20 °C in amber vials until use.

### 4.3. Phytochemistry Analysis by HPLC-ESI-QTOF-MS/MS

Dried extracts were reconstituted with EtOH:H_2_O_2_ (50:50, *v*:*v*) in a final concentration of 5 mg extract/mL. Samples were vortexed for 1 min, subjected to ultrasonic treatment for 10 min, and subsequently filtered through a 0.22 μm membrane before being transferred into vials for HPLC-ESI-QTOF-MS/MS analysis. The chromatographic analyses were carried out on an Agilent 1290 HPLC system coupled to a QTOF mass spectrometer (model G6530C UHD, Agilent Technologies, Santa Clara, CA, USA). Separation was achieved in reversed-phase mode using an ACQUITY UPLC BEH Shield RP18 column (2.1 × 150 mm, 1.7 μm, 130 Å; Waters Corporation, Milford, MA, USA) maintained at 60 °C. The mobile phases consisted of water with 0.1% formic acid (solvent A) and acetonitrile (solvent B), applying the following gradient: 0.0 min [A:B, 95:5], 5.0 min [90:10], 18.0 min [15:85], 24.0–25.5 min [0:100], and 26.5–32.5 min [95:5]. The injection volume was 5 μL, and the flow rate was set to 0.4 mL min^−1^. Mass spectrometry data were acquired in negative electrospray ionization mode (ESI–) under data-dependent acquisition mode (DDA), covering an *m*/*z* range of 50–1200 at a rate of 3 and 10 spectra per second for MS1 and MS2 scans, respectively. DDA analyses were performed using collision energies of 10, 20, and 60 eV. N_2_ served as both the nebulizing and drying gas. The optimized source conditions were as follows: drying gas temperature, 200 °C; gas flow rate, 10 L min^−1^; nebulizer pressure, 20 psig; desolvation temperature, 350 °C; capillary voltage, 4 kV; nozzle voltage, 500 V; and nebulization pressure, 2 bar. Continuous mass calibration was achieved through the infusion of a reference solution containing purine (*m*/*z* 112.985587) and HP-921 (*m*/*z* 1033.988109). The raw data files (.d format) were first converted using MSConvert (https://proteowizard.sourceforge.io/, accessed on 8 May 2025) and subsequently processed with MZmine 4.7.29 for data pre-processing [64]. The workflow included background noise removal, chromatogram construction (ADAP algorithm), chromatogram deconvolution, isotope grouping, and alignment. Noise thresholds were set at 2.5 × 10^3^, for MS1 and 2 × 10^2^, for MS2 scans. ADAP chromatograms were generated with a minimum consecutive scan of 6, a minimum highest intensity of 5 × 10^4^, and an *m*/*z* tolerance of 0.005 Da. Deconvolution was carried out using the local minimum feature resolver under the following settings: chromatographic threshold: 85%; minimum search range: 0.05 min; minimum absolute height: 5 × 10^4^; and min ratio of peak top/edge: 1.70. MS/MS fragmentation scans were paired with the corresponding deconvoluted peaks using a MS1 to MS2 precursor tolerance of 0.005 Da. For isotope grouping, the parameters applied were an *m*/*z* tolerance of 0.005 Da, a retention time tolerance of 0.1 min, a monotonic shape option, and a maximum charge state of 2. The alignment of chromatograms was performed using the Join Aligner algorithm, with tolerances of 0.005 Da (*m*/*z*) and 0.2 min (RT). Molecular formula prediction and structural elucidation were carried out using Sirius 6.3.2 [65], and the tentative identifications were verified by comparison with literature data. This software was used for the annotation of phytochemical compounds in the extracts using in silico prediction methods. In parallel, the “Library Search” function of the MZMine software (https://github.com/mzmine/mzmine/releases/latest, accessed on 8 May 2025) was used for compound annotation by similarity comparison with MS/MS spectra available in MS repositories (MassBank, MoNA).

### 4.4. Pharmacokinetic Analysis Using Computational Tools

Nine candidate compounds were drawn in ChemDraw 16.0, converted to canonical SMILES, and profiled with SwissADME (https://www.swissadme.ch/index.php, accessed on 8 May 2025) [66] to generate a unified ADME readout predicting their key in vivo pharmacokinetics.

### 4.5. Evaluation of the Cytotoxic Effects of Crocus sativus Corm Extracts on SW480 and T84 Colon Cancer Cells

#### 4.5.1. Preparation of the Cell Culture

Colon cancer cell lines SW480 and T84 were purchased from American Type Culture Collection (ATCC, Manassas, VA, USA), cultured in Dulbecco’s Modified Eagle Medium (DMEM, Gibco, Life Technologies, Madrid, Spain) supplemented per liter with 10% fetal bovine serum (FBS, Gibco, Life Technologies, Spain), 1% penicillin/streptomycin (100 U/mL penicillin and 100 µg/mL streptomycin, Sigma, Madrid, Spain), and 1% L-glutamine (2 mM) (Gibco, Life Technologies, Spain). DMEM provides an array of essential nutrients necessary for the growth and maintenance of human cells. The cells were maintained at 37 °C in a humidified atmosphere containing 5% CO_2_ to ensure their viability.

SW480 (primary colorectal adenocarcinoma) and T84 (metastatic colorectal carcinoma) were chosen to represent different stages and barrier properties of CRC, providing a broader view of the extracts’ activity spectrum.

#### 4.5.2. Crystal Violet Assay

Cell viability was evaluated using the crystal violet test following treatment of the cells with corm extracts (HEES, HEEF) at doses of 10, 50, 100, and 250 µg/mL for 24, 48, and 72 h in 12-well microplates. Stock solutions of the dried extracts were prepared in bidistilled water and filtered before dilution into complete culture medium immediately prior to treatment. The extracts were completely soluble at all tested concentrations, and no visible precipitation was detected. After treatment, the culture medium was removed and 40 µL of crystal violet solution (0.75 g of crystal violet in 120 mL of distilled water and 30 mL of methanol) was added to each well after the culture medium was removed. The crystal violet solution was removed after 20 min of gentle shaking, and each well was washed three times with 1 mL of distilled water, allowing approximately 30 s of contact per wash before aspiration. The plate was then air-dried at room temperature, and 160 μL of methanol was added to each well to dissolve the bound dye. Absorbance at 570 nm was used to calculate the proportion of living adhering cells [67]. No reference positive control drug was included; therefore, the IC50 values are interpreted primarily as relative comparisons between extracts within this experimental setup.

#### 4.5.3. Total Protein Extraction

Proteins were isolated from T84 and SW480 cells in RIPA Lysis Buffer (150 mM NaCl, 50 mM Tris-HCl, 1% Nonidet P-40, 0.5% sodium deoxycholate, and 0.1% sodium dodecyl sulfate (SDS)) with 1% Triton X-100 and 1% protease inhibitor cocktail (Thermo Fisher Scientific, Waltham, MA, USA). The samples were homogenized using a Teflon pestle while ensuring that the temperature was maintained at 4 °C throughout all procedures. Homogenates were then centrifuged at 13,000 g for 15 min at 4 °C. The clear supernatant was carefully transferred to a new microcentrifuge, and aliquots of total protein lysate were stored at −80 °C. Quantification of total isolated proteins of samples was determined by the Bradford method. Bovine serum albumin (BSA) was used as a standard [68].

#### 4.5.4. Western Blot Analysis

Protein extracts (50 µg per sample) were separated by SDS–polyacrylamide gel electrophoresis (SDS-PAGE) using a tricine-containing running buffer (0.1 M Tris-HCl, 0.1% SDS, 0.1 M Tricine) to achieve better band resolution. Proteins were subsequently transferred onto nitrocellulose membranes (Bio-Rad Trans-Blot SD, Bio-Rad Laboratories, Hercules, CA, USA). The membranes were then blocked at room temperature for 1 h using a blocking buffer composed of phosphate-buffered saline (PBS: 137 mM NaCl, 2.7 mM KCl, 10 mM Na_2_HPO_4_, and 1.8 mM KH_2_PO_4_) supplemented with 1% Tween-20 and 5% non-fat dry milk.

Following blocking, the membranes were incubated overnight at 4 °C with primary antibodies. Mouse anti-P53 (DO-1 cat#SC-126; Santa Cruz Biotechnology, Dallas, TX, USA) and mouse anti-caspase-9 (cat#SC-133109; Santa Cruz Biotechnology, Dallas, TX, USA) were used at a dilution of 1:1000 in blocking buffer. To ensure equal protein loading, mouse anti-β-actin (cat#SC-81178; Santa Cruz Biotechnology, Dallas, TX, USA) was applied at the same dilution.

After the primary antibody incubation, the membranes were washed three times (15 min each) with PBS containing 1% Tween-20 (PBS-T) to remove unbound antibodies. The membranes were then treated for 2 h at room temperature with horseradish peroxidase (HRP)-conjugated secondary antibodies (Sigma-Aldrich, Madrid, Spain) at a 1:2000 dilution.

Subsequently, the membranes were washed three times (15 min each) with PBS-T, and protein signals were visualized using the Clarity Western ECL detection kit (Bio-Rad, Santiago de Compostela, Spain) per the manufacturer’s instructions. Signal intensities were captured using the Kodak Image Station 4000 MM Pro Molecular Imaging system (Eastman Kodak, Rochester, NY, USA) and analyzed with ImageJ software (version 1.33, National Institutes of Health, Bethesda, MD, USA). All signals were normalized to β-actin, and experiments were performed in duplicate for reproducibility. Because a pharmacological positive control treatment was not included, the Western blots are interpreted as relative changes versus untreated controls; nevertheless, internal standardization was ensured by equal protein loading, parallel processing, and β-actin normalization [68].

No reference positive control was included in the Western blot experiments; untreated cells served as the internal reference, and this is noted as a limitation.

### 4.6. Molecular Docking with PyRx and AutoDock Vina

**Figure 12:**
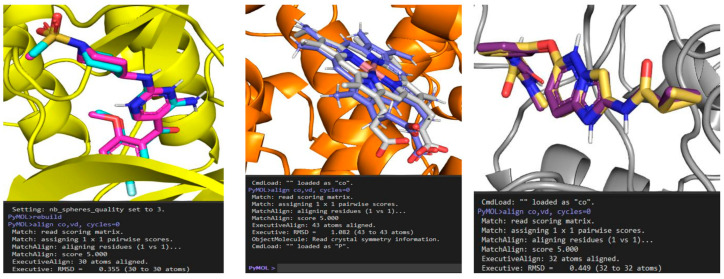
Docking protocol validation by redocking of co-crystal ligands. Overlay of native vs. redocked poses for 2FVD (RMSD 0.355 Å), 5KIR (1.082 Å), and 3VO3 (0.449 Å).

Nine candidate ligands were converted to SMILES in PyRx 0.9.8, assembled into a multi-ligand SDF, and energy-minimized with UFF (500 conjugate-gradient steps). Lowest-energy conformers were exported as PDBQT files with Gasteiger charges and auto-assigned rotatable bonds. Receptors (1NW9, 2FVD, 5KIR, 1M14, 7WXX, 1TUP, 1JPW, 3VO3) were prepared in AutoDock Tools 1.5.7 by removing crystallographic waters/non-essential heteroatoms, adding polar hydrogens and Kollman charges, and retaining essential cofactors/ions; where applicable, only chain A was kept to reduce system size. Docking was performed with AutoDock Vina 1.1.2 via PyRx (exhaustiveness = 8) [69]. Protocol fidelity was confirmed by redocking native co-crystal ligands (Figure 12), yielding RMSD = 0.355 Å (2FVD), 1.082 Å (5KIR), and 0.449 Å (3VO3), well within the ≤2.0 Å benchmark, while blind docking was applied to the remaining five proteins (1NW9, 1M14, 7WXX, 1TUP, 1JPW). Binding poses and scores were analyzed in Discovery Studio 2024 and PyMOL 2.5 to map interaction networks and rationalize ligand stabilization.

### 4.7. Implementation of Molecular Dynamics Simulations Using GROMACS

Molecular dynamics (MD) simulations were carried out with GROMACS 2021.3 [70]. Protein topologies were prepared using gmx pdb2gmx with the AMBER99SB-ILDN force field, adding missing hydrogens and adjusting protonation states as required. The ligand was parameterized separately to produce validated .itp and .gro files and then combined with the protein to assemble the full complex. Systems were solvated in a cubic TIP3P water box, neutralized with counterions, and energy-minimized by steepest descent. Equilibration proceeded under NVT and then NPT to stabilize temperature and pressure, followed by a 100 ns production run that recorded coordinates and velocities at regular intervals to assess stability, conformational dynamics, and key protein–ligand interactions under near-physiological conditions.

### 4.8. MM/GBSA Calculation

Binding free energies were evaluated by MM/GBSA with AmberTools 23 (MMPBSA.py, parallel mode). For each system, 100 snapshots were taken every 0.4 ns from the 60–100 ns portion of the GROMACS trajectory. The HCT Generalized Born model (igb = 5) was used with dielectric constants ε_in = 1.0 and ε_out = 80.0 and an ionic strength of 0.15 M; the nonpolar term was derived from the solvent-accessible surface area. Per-residue decomposition (idecomp = 1) was enabled to separate van der Waals, Coulombic, polar (GB), and nonpolar (surface) contributions, and temporary files were removed after execution (keep_files = 0).

### 4.9. Statistical Analysis

Data are expressed as mean ± standard error of the mean (SEM). For each condition, three independent biological experiments were performed, each containing at least three technical replicate wells when applicable. The means of the biological replicates (*n* = 3) were analyzed using one-way ANOVA followed by Tukey’s multiple comparison post hoc test to compare all treatment groups. Statistical significance was set at *p* < 0.05, *p* < 0.01, and *p* < 0.001, and analyses were performed with GraphPad Prism 8.0.

## 5. Conclusions

Within the limitations of this study (single harvest batches collected in different years; real-world storage without full environmental control; semi-quantitative HPLC-ESI-QTOF-MS/MS profiling; and no reference anticancer drug control in the viability assay), the stored-corm extract (HEES) displayed a distinct, more oxidized secondary metabolite profile and stronger in vitro antiproliferative effects than the fresh-corm extract (HEEF). The in silico screen suggests that different constituents may plausibly engage distinct cancer-relevant targets (multitarget potential at the constituent level), but mixture-level synergy/competition was not quantified. Overall, these findings nominate saffron corms, particularly after storage under the tested conditions, as a potentially valuable source of secondary metabolites (notably anthraquinone- and carotenoid-related compounds) for follow-up work. Future studies should include controlled storage time-course experiments with biological replicates, targeted quantification using authentic standards, inclusion of reference anticancer drugs, expanded mechanistic assays, and in vivo validation.

## Figures and Tables

**Table 1:** SwissADME Descriptors, Rule-of-Five/Veber Compliance, bioavailability score, and predicted CYP liabilities for the 9 major compounds.

Molecules	1	2	3	4	5	6	7	8	9
Molecular WEIGHT(g/mol)	206.15	188.22	328.27	314.25	298.25	328.27	342.43	312.27	192.12
H-bond acceptors	5	4	7	7	6	7	4	6	7
H-bond donors	2	2	3	4	3	3	1	2	4
Rotatable bonds	1	8	2	1	1	2	9	2	5
TPSA (Å^2^)	87.74	74.6	121.13	132.13	111.9	121.13	63.6	100.9	132.13
CLogP	1.1	1.49	1.72	1.43	1.81	1.87	1.87	2.28	−1.51
Lipinski: violations	0	0	0	0	0	0	0	0	0
Veber: violations	0	0	0	0	0	0	0	0	0
Bioavailability score	0.56	0.85	0.56	0.56	0.56	0.56	0.56	0.55	0.56
CYP2D6 inhibitor	No	No	No	No	No	No	No	No	No
CYP3A4 inhibitor	No	No	Yes	Yes	No	Yes	Yes	Yes	No

**Table 2:** IC_50_ values for cell viability after treatment with *Crocus sativus* corm extracts determined by crystal violet assay. The mean ± standard error of three measurements is used to present the results.

Colon Cancer Cells	Corm Extract	IC_50_ (µg/mL) (24 h)	IC_50_ (µg/mL) (48 h)	IC_50_ (µg/mL) (72 h)
SW480	HEES	34.85 ± 3.35	17.1799 ± 0.883	23.83 ± 0.372
	HEEF	186.60 *** ± 1.14	35.38 ± 5.44	40.913 ± 5.081
T84	HEES	38.140 ± 1.257	48.60 ± 2.410	37.05 ± 2.623
	HEEF	44.26 ± 9.722	45.74 ± 3.055	43.461 ± 6.790

HEEF: hydroethanolic extract of fresh corms; HEES: hydroethanolic extract of stored corms. *** (*p* < 0.001), significantly different from HEES within the same cell line and at the same time point (one-way ANOVA followed by Tukey’s multiple comparison test).

**Table 3:** Docking binding energies (kcal/mol) of the 9 major compounds against eight cancer-relevant protein targets.

Sample Name	1NW9	2FVD	5KIR	1M14	7WXX	1TUP	1JPW	3VO3
7-Hydroxycoumarin-6-carboxylic acid	−6.7	−7.2	−7.5	−6.2	−6.4	−7.1	−5.8	−7.5
Azelaic acid	−5.0	−5.2	−5.9	−4.8	−5.2	−4.8	−4.1	−6.1
Rhamnalpinogenin	−7.2	−8.6	−7.9	−8.2	−6.9	−7.7	−6.8	−7.2
Endocrocin	−7.7	−8.6	−8.2	−8.2	−7.5	−7.6	−6.6	−6.7
3,8-Dihydroxy-1-methylanthraquinone-2-carboxylic acid	−7.2	−8.9	−8.7	−8.3	−6.9	−8.0	−7.0	−7.8
Cinnalutein	−7.0	−8.7	−8.1	−8.3	−7.6	−7.7	−6.8	−7.0
Crocetin	−6.9	−7.6	−7.3	−6.6	−6.4	−6.8	−5.9	−9.8
Aloesaponarin I	−6.9	−8.7	−8.6	−7.6	−6.9	−7.6	−6.6	−8.5
Citric acid	−5.3	−4.6	−6.0	−5.0	−5.7	−4.9	−4.5	−5.4

**Table 4:** MM/GBSA binding free-energy components for the three complexes (kcal·mol^−1^).

	Kcal/mol		
Molecules	1-Me-AQ-2-COOH--CDK2	1-Me-AQ-2-COOH--COX-2	Crocetin--VEGFR2
ΔE_vdW_ (vdW interactions)	−29.93	−39.94	−50.62
ΔE_ele_ (electrostatic)	−34.23	−21.75	−7.36
ΔG_GB_ (polar solvation)	47.12	36.64	21.21
ΔG_surf_ (nonpolar solvation)	−4.42	−4.39	−7.73
ΔG_gas_ (gas phase)	−64.16	−61.69	−57.98
ΔG_solv_	42.71	32.25	13.48
ΔG_binding_	−21.45	−29.44	−44.51

## Data Availability

The original contributions presented in this study are included in the article. Further inquiries can be directed to the corresponding author.

## Abbreviations

The following abbreviations are used in this manuscript:

ATCC	American Type Culture Collection
CRC	Colorectal cancer
DMEM	Dulbecco’s Modified Eagle Medium
FBS	Fetal bovine serum
SDS	Sodium dodecyl sulfate
SDS-PAGE	Sodium dodecyl sulfate–polyacrylamide gel electrophoresis
PBS	Phosphate-buffered saline
BSA	Bovine serum albumin
RIPA	RadioImmunoPrecipitation Assay
HRP	Horseradish peroxidase
HEEF	Hydroethanolic extract of fresh corms
HEES	Hydroethanolic extract of stored corms
HPLC	High-performance liquid chromatography
ESI	Electrospray ionization
QTOF	Quadrupole time-of-flight
MS	Mass spectrometry
MS/MS	Tandem mass spectrometry
DDA	Data-dependent acquisition
ADAP	Automated Data Analysis Pipeline
RT	Retention time
EtOH	Ethanol
ADME	Absorption, distribution, metabolism, excretion
COX-2	Cyclooxygenase-2
VEGFR2	Vascular endothelial growth factor receptor-2
SW480	Human colorectal adenocarcinoma cell line
T84	Human colorectal carcinoma cell line
RPM	Revolutions per minute
HCT	Hawkins–Cramer–Truhlar model
MM/GBSA	Molecular mechanics/generalized Born surface area
MD	Molecular dynamics
GROMACS	Groningen Machine for Chemical Simulations
TIP3P	Transferable Interaction Potential 3-Point water model
RMSD	Root-mean-square deviation
ANOVA	Analysis of variance
p53	Tumor protein p53
Caspase-9	Cysteine-aspartic acid protease-9
1-Me-AQ-2-COOH	3,8-Dihydroxy-1-methylanthraquinone-2-carboxylic acid

## Appendix A

**Table A1:** Phytochemical analysis of hydroethanolic extracts of stored (HEES) and fresh (HEEF) *Crocus sativus* corms.

Number	RT	Row *m*/*z*	Adduct_Type	Name	Phytochemical Class	Molecular Formula	MS/MS Fragments	HEES (%)	HEEF (%)
1	0.68	377.0863	[M − H]^−^	Sucrose	Sugar	C_12_H_22_O_11_		0	0.854
2	0.68	165.0402	[M − H]^−^	Arabinoic acid	Sugar	C_5_H_10_O_6_	59/75/129/147	1.24	0.42
3	0.68	215.0326	[M + Cl]^−^	Fructose	Sugar	C_6_H_12_O_6_	59/71/89	0.28	2.36
4	0.74	119.0338	[M − H]^−^	Erythrose	sugar	C_4_H_8_O_4_	59	0.00	0.41
5	0.76	133.0141	[M − H]^−^	Malate	Dycarboxilic acid	C_4_H_6_O_5_	71/73/89/115	2.32	1.62
6	0.77	173.0072	[M − H]^−^	Arginine	Amino acid	C_6_H_14_N_4_O_2_	131/156	0.36	0.00
7	0.82	191.0188	[M − H]^−^	Citric acid	Tricarboxilic acid	C_6_H_8_O_7_	57/67/87/111	6.04	12.46
8	0.98	253.0900	[M + FA − H]^−^	Ethyl glucoside	Glycoside	C_8_H_16_O_6_		0.21	0.99
9	0.98	115.0025	[M − H]^−^	Maleic acid	Organic acid	C_4_H_4_O_4_		0.64	0.52
10	1.02	173.0093	[M − H]^−^	Aconitic acid	Organic acid	C_6_H_6_O_6_	85/111	0.67	1.13
11	1.05	147.0304	[M − H]^−^	Citramalate	Hydroxy fatty acid	C_5_H_8_O_5_	85/87/103/129	0.23	0.20
12	1.07	117.0168	[M − H]^−^	Succinic acid	Organic acid	C_4_H_6_O_4_	55/73/99	0.74	0.50
13	1.19	129.0167	[M − H]^−^	Itaconic acid	Dycarboxilic acid	C_5_H_6_O_4_	85/129	0.87	0.39
14	2.34	154.0145	[M − H]^−^	Unknown		C_6_H_5_NO_4_		0.65	0.00
15	3.51	181.0149	[M − H]^−^	Hydroxyisophthalic acid	Phenolic compound	C_8_H_6_O_5_	65/93/137	0.28	0.00
16	3.59	137.0245	[M − H]^−^	Protocatechuic aldehyde	Phenolic compound	C_7_H_6_O_3_	108/109/119/	0.00	0.45
17	4.21	175.0613	[M − H]^−^	Isopropylmalic acid	Organic acid	C_7_H_12_O_5_	85/113/115/131/157	0.38	1.21
18	4.48	227.0206	[M + FA − H]^−^	Hydroxyphtalic acid	Phenolic compound	C_8_H_6_O_5_	183	1.60	0.17
19	6.33	153.0197	[M − H]^−^	Dyhydroxybenzoic acid	Phenolic compound	C_7_H_6_O_4_	109/135	0.36	0.00
20	7.30	211.0258	[M + FA − H]^−^	Phthalic acid	Organic acid	C_8_H_6_O_4_	80/108/123/167	2.07	0.56
21	7.70	163.0383	[M − H]^−^	Coumaric acid	Phenolic compounds	C_9_H_8_O_3_	93/119	0.00	0.34
22	7.78	223.0261	[M − H]^−^	Dehydrochorismic acid	Aromatic acid	C_10_H_8_O_6_	107/135/179	0.42	0.93
23	7.99	249.0774	[M + Cl]^−^	Citrinin	Polyketide	C_13_H_14_O_5_	175/187/205	0.19	0.18
24	8.76	375.1095	[M + FA − H]^−^	Furanoid lignan derivative	Lignan	C_18_H_18_O_6_		1.31	1.54
25	8.87	239.0204	[M + FA − H]^−^	Phthalic acid derivative	Organic compounds	C_9_H_6_O_5_	Level 3	2.28	0.74
26	8.93	211.0254		Unknown	Unknown	Unknown		0.73	0.00
27	9.06	235.0624	[M − H]^−^	Orthosporin	Isocomarin	C_12_H_12_O_5_	191	0.29	0.70
28	9.12	261.0080	[M − H]^−^	Acetylphenol sulfate	Phenolic compound	C_9_H_10_O_7_S	138/166/181	0.37	1.47
29	9.16	251.0204	[M + FA − H]^−^	7-Hydroxycoumarin-6-carboxylic acid	Hydroxycoumarin derivative	C_10_H_6_O_5_	135/163/207/235	1.10	2.42
30	9.19	361.0960	[M − H]^−^	Secosterigmatocystin	Hydroxyanthraquinone derivative	C_18_H_18_O_8_	228/240/283/301	0.72	0.00
31	9.26	475.0919	[M + FA − H]^−^	Anthracenecarboxylic acid derivative	Anthraquinone	C_21_H_18_O_10_	Level 3	0.65	0.36
32	9.39	187.0967	[M − H]^−^	Azelaic acid	Fatty acids	C_9_H_16_O_4_	97/125/143/169	1.40	2.89
33	9.39	623.1647	[M − H]^−^	Hydroxycinnamic acid glycoside derivative	Phenolic compound	C_28_H_32_O_16_	Level 3	0.18	0.85
34	9.44	287.0571	[M − H]^−^	Dyhydrokaempferol	Flavonoid	C_15_H_12_O_6_	125/243/259	1.37	0.00
35	9.49	329.1022	[M + FA − H]^−^	Glypallichalcone isomer	Flavonoid	C_17_H_16_O_4_	135/147/165	0.91	1.76
36	9.55	327.0528	[M − H]^−^	Rhamnalpinogenin	Anthracenecarboxylic acid derivative	C_17_H_12_O_7_	212/240/268/283	3.48	0.00
37	9.57	433.1520	[M + FA − H]^−^	Medioresinol	Lignans	C_21_H_24_O_7_	373	0.20	0.00
38	9.65	235.0613	[M − H]^−^	Isocoumarin derivative	Isocoumarins	C_12_H_12_O_5_	Level 3	0.31	0.55
39	9.70	221.0795	[M − H]^−^	Hydroxycinnamic acid derivative	Phenylpropanoids	C_12_H_14_O_4_	106/178/206	1.54	0.00
40	9.90	281.0318	[M + FA − H]^−^	Penicipyran C	Hydroxycoumarin derivative	C_11_H_8_O_6_	83/135/150/209/237	1.27	2.21
41	10.00	313.0362	[M − H]^−^	Endocrocin	Porphyrins	C_16_H_10_O_7_	269	2.86	1.07
42	10.20	343.0480	[M + FA − H]^−^	Anthracenecarboxylic acid derivative	Anthraquinones	C_16_H_10_O_6_	256/284/299	0.91	0.32
43	10.29	373.0542	[M + FA − H]^−^	Anthracenecarboxylic acid derivative	Anthraquinones	C_17_H_12_O_7_	Level 3	0.00	0.00
44	10.32	265.0356	[M + FA − H]^−^	Hydroxycoumarin derivative		C_11_H_8_O_5_	178/206/234	0.00	1.59
45	10.33	287.0546	[M − H]^−^	Monodictyphenone	Hydroxybenzoic acid derivative	C_15_H_12_O_6_	91/135/177/243	0.00	0.00
46	10.43	493.1519	[M − H]^−^	Isoamericanol derivative	Furanoid lignan derivative	C_27_H_26_O_9_	327	0.34	0.49
47	10.43	311.0206	[M − H]^−^	Anthracenecarboxylic acid derivative	Anthraquinones	C_16_H_8_O_7_	183/211/239/267	0.60	0.25
48	10.44	309.1327		Unknown		C_15_H_20_O_4_		0.16	0.00
49	10.58	447.2585	[M − H]^−^	Fatty acyl glycoside derivative	Fatty acids	C_22_H_40_O_9_	Level 3	1.01	1.22
50	10.65	295.0479	[M − H]^−^	Hydroxybenzoic acid derivative	Phenolic compound	C_13_H_12_O_8_	Level 3	0.73	0.78
51	10.71	311.0215	[M − H]^−^	Anthracenecarboxylic acid derivative	Anthraquinones	C_16_H_8_O_7_	211/239/267	0.23	0.00
52	10.72	279.1241	[M + Cl]^−^	Xylaric acid D	Terpene lactone derivative	C_12_H_20_O_5_	147/177/217	0.75	0.00
53	10.87	313.0356	[M − H]^−^	Variolaric acid	Anthraquinones	C_16_H_10_O_7_	224/225/241/269	2.08	1.02
54	10.92	809.4347	[M − H]^−^	Dianoside A	Triterpene glycoside derivative	C_42_H_66_O_15_	89/113/119/585	0.42	0.80
55	11.82	329.2335	[M − H]^−^	FA 18:1 + 3O	Oxidized fatty acid	C_18_H_34_O_5_	99/139/171/211/229	2.22	4.16
56	12.10	530.2755		Unknown		Unknown		0.35	0.00
57	12.27	617.0712	[M − H]^−^	Anthracenecarboxylic acid derivative	Anthraquinone	C_34_H_18_O_12_	Level 3	0.74	0.72
58	12.29	297.0415	[M − H]^−^	3,8-Dihydroxy-1-methylanthraquinone-2-carboxylic acid	Anthracenecarboxylic acid derivative	C_16_H_10_O_6_	225/253	6.42	4.82
59	12.42	277.0355	[M + FA − H]^−^	Hydroxycoumarin derivative	Furanocoumarins	C_12_H_8_O_5_	162/234/262	0.00	0.00
60	12.43	329.2357	[M − H]^−^	Trihydroxyoctadecenoic acid	Fatty acids	C_18_H_34_O_5_	Level 3	0.19	0.25
61	12.54	295.0266	[M − H]^−^	Anthracenecarboxylic acid derivative	Anthraquinone	C_16_H_8_O_6_	Level 3	1.26	1.46
62	12.57	213.1136	[M − H]^−^	Hexylitaconic Acid	Dicarboxylic acid	C_11_H_18_O_4_	Level 3	1.22	0.00
63	12.72	313.0359	[M − H]^−^	Laccaic acid D isomer	Anthracenecarboxylic acid derivative	C_16_H_10_O_7_	269	0.19	0.00
64	12.85	327.0519	[M − H]^−^	Cinnalutein	Anthracenecarboxylic acid derivative	C_17_H_12_O_7_	212/240/268/283	9.35	8.17
65	12.94	565.1156	[M − H]^−^	Flavonoid C-glycoside derivative	Flavonoid	C_25_H_26_O_15_	Level 3	1.08	0.18
66	12.95	595.1259	[M + FA − H]^−^	Isoflavonoid O-glycoside derivative	Flavonoid	C_25_H_26_O_14_	238/268/282/312	0.64	0.21
67	13.05	343.1784	[M − H]^−^	tricarboxylic acid derivative	Tricarboxylic acid	C_17_H_28_O_7_	Level 3	1.18	0.00
68	13.42	327.1436	[M − H]^−^	Crocetin	Carotenoid	C_20_H_24_O_4_	Level 3	2.38	0.00
69	13.43	495.0582	[M − H]^−^	7-Phloroeckol	Methylated flavonoid derivative	C_24_H_16_O_12_	Level 3	0.00	0.00
70	13.52	293.1773	[M − H]^−^	Gingerol	Phenolic compound	C_17_H_26_O_4_	148/177/205/221	0.55	1.99
71	13.54	269.0467	[M + FA − H]^−^	1-Hydroxyanthraquinone	Hydroxyanthraquinone derivative	C_14_H_8_O_3_	226/239	0.33	0.18
72	13.91	325.1676	[M − H]^−^	Dicarboxylic acid derivative	Fatty acid	C_17_H_26_O_6_	Level 3	0.43	0.00
73	14.35	311.0574	[M − H]^−^	Aloesaponarin I	Tetracenequinone derivative	C_17_H_12_O_6_	224/253/267	2.79	4.06
74	14.42	253.0515	[M − H]^−^	Chrysophanol	Hydroxyanthraquinone derivative	C_15_H_10_O_4_	182/225	2.39	2.27
75	14.45	313.2389	[M − H]^−^	FA 18:1 + 2O	Oxidized fatty acid	C_18_H_34_O_4_	Level 3	0.83	1.14
76	14.66	313.2388	[M − H]^−^	FA 18:1 + 2O	Oxidized fatty acid	C_18_H_34_O_4_	Level 3	2.88	0.96
77	14.83	327.1821	[M − H]^−^	Spiculirsporic acid	Tricarboxylic acid derivative	C_17_H_28_O_6_	Level 3	0.19	0.00
78	14.85	283.0620	[M − H]^−^	Physcion	Anthraquinone derivative	C_16_H_12_O_5_	197/212/240	0.62	0.81
79	14.85	341.0681	[M − H]^−^	Methylisoflavone derivative	Flavonoid	C_18_H_14_O_7_	Level 3	0.64	0.99
80	15.49	315.2553	[M − H]^−^	Dihydroxyoctadecanoic acid	Hydroxylated fatty acid	C_18_H_36_O_4_	Level 3	1.97	0.71
81	15.74	265.1486	[M − H]^−^	Lauryl sulfate	Sulfuric acid monoester derivative	C_12_H_26_O_4_S	Level 3	0.59	0.73
82	16.26	295.2285	[M − H]^−^	Hydroxyoctadecadienoic acid	Fatty acid	C_18_H_32_O_3_	Level 3	1.88	2.78
83	16.99	561.3289	[M + FA − H]^−^	MGMG 18:2	Glycosyldiradylglycerols	C_27_H_48_O_9_	Level 3	0.37	0.80
84	18.90	277.2181	[M − H]^−^	Linolenic acid	Fatty acid	C_18_H_30_O_2_	Level 3	0.84	2.26
85	19.11	271.2276	[M − H]^−^	Hydoxy hexadecanoate	Fatty acid	C_16_H_32_O_3_	Level 3	1.09	1.32
86	19.87	379.1593		Unknown		Unknown		1.15	1.90
87	19.88	279.2340	[M − H]^−^	Rumenic acid	Fatty acid	C_18_H_32_O_2_	Level 3	2.06	3.03
88	20.96	355.1593		Unknown		Unknown		0.77	1.22
89	20.96	255.2335	[M − H]^−^	Palmitic acid	Fatty acid	C_16_H_32_O_2_	Level 3	0.71	1.10
90	21.14	281.2494	[M − H]^−^	Oleic acid	Fatty acid	C_18_H_34_O_2_	Level 3	1.53	2.16
91	22.77	383.1868		Unknown		Unknown		0.43	0.88
